# Measuring dysfunctional interpersonal beliefs: validation of the Interpersonal Cognitive Distortions Scale among a heterogeneous German-speaking sample

**DOI:** 10.1186/s12888-023-05155-3

**Published:** 2023-09-27

**Authors:** Lukas Kirchner, Matthias Kloft, Beatriz Arias Martín, Max Berg, Paria Anjedanimoghadamaraghi, Leonora Schäfer, Winfried Rief

**Affiliations:** 1 Department of Psychology, Clinical Psychology and Psychotherapy, Philipps-University of Marburg, Gutenbergstraße 18, 35037 Marburg, Germany; 2 Department of Psychology, Psychological Methods, Philipps-University of Marburg, Marburg, Germany; 3 Department of Psychology, Psychological Diagnostics, Philipps-University of Marburg, Marburg, Germany

**Keywords:** Expectation, Belief, Cognitive model, Mental health, Psychopathology, Interpersonal risk factor

## Abstract

**Backround:**

Dysfunctional interpersonal beliefs (DIBs) are a key symptom domain in numerous mental disorders. Because DIBs exert a strong influence on social experience and behavior, they play an important role in a mental disorder's development and progression. To date, only the Interpersonal Cognitive Distortions Scale (ICDS) captures DIBs independently of *specific* disorders, populations, or contexts. The present study's aim was to psychometrically evaluate and validate a German translation of the ICDS.

**Methods:**

The ICDS was administered along with indicators of convergent (rejection sensitivity, depressive expectations, interpersonal trust, interpersonal problems, perceived social support), discriminant (self-efficacy, perseverative negative thinking, optimism), and clinical validity (psychopathology, perceived stress, well-being) to a pooled sample incorporating non-clinical (*N* = 114) and clinical (*N* = 94) participants.

**Results:**

An exploratory factor analysis (EFA) suggested a five-factor solution (factor loadings: .44 to .85). Correlational analyses demonstrated acceptable convergent (*ρ* = -.29 to -.35, *ρ* = .27 to .59), suboptimal discriminant (*ρ* = -.27 to -.38, *ρ* = .52), and acceptable clinical validity (*ρ* = -.21, *ρ* = .36 to .44) at the total-scale level. However, results at the subscale level were mixed and required nuanced interpretation. Likewise, internal consistency was acceptable at the total-scale level (α = .76), but ranged from good to poor at the subscale level (α = .61 to .80). DIBs mediated the negative relationship between mental disorder onset and psychopathology levels.

**Discussion:**

Our results imply DIBs' relevance to mental health and related outcomes. When working with the ICDS’s German version, we recommend employing only the “insecurity” subscale, as this was the only scale revealing acceptable psychometric properties. Future studies should improve the construct validity of the ICDS (and its subscales), e.g., by adding more items to the respective subscales and further classes of DIBs.

**Supplementary Information:**

The online version contains supplementary material available at 10.1186/s12888-023-05155-3.

## Introduction

Dysfunctional interpersonal beliefs (DIBs), such as expecting social rejection (e.g., “people will reject me”) or interpersonal exploitation (e.g., “people will harm me”), have been proposed to constitute important characteristics of various mental disorders. DIBs are especially common in psychosis [1], social phobia [2], post-traumatic stress disorder [3], depression [4], and borderline personality disorder [5]. Even across short time periods, DIBs have been shown to encourage the development and maintenance of depressive symptoms [6].

In line with this notion, numerous findings from general, clinical, and social psychology suggests that DIBs play an important role in aberrant anticipation [7], attention [8], perception [9], memorization [10], and interpretation of social stimuli [11], as well as in distorted social behavior [12]. Thus, they constitute a trans-diagnostic risk marker for psychopathology [13, 14]. For instance, “perceived burdensomeness”, or the belief of being a burden to important others, turned out to be strongly related to symptoms of depression and suicide attempts [15].

Other lines of research also suggest that DIBs exert negative effects on mental health and well-being. In couple research, for example, there is evidence that DIBs undermine dyadic coping, quality of life, and satisfaction within romantic relationships [16–20].

Moreover, there is evidence that DIBs are particularly important when it comes to the transition from transient mental conditions to more chronic and treatment-resistant forms of mental disorders. This is particularly evident in the etiology of personality disorders, where DIBs often develop as a result of social trauma [21] and lead, in turn, to social misperceptions and deviant social behavior [22]. As a consequence, social misperceptions and deviant social behavior could raise the risk of re-experiencing social trauma in the future [23]. This might eventually lead into a vicious circle of social maladaptation and re-traumatization through aberrant belief systems.

Finally, DIBs are also a serious problem in the effective treatment of mental disorders, e.g., when establishing therapeutic collaboration, as they can negatively influence the perception of the therapeutic relationship and thus the therapeutic process [24]. Think, for example, of patients with social phobia who do not dare to tell their therapist that they did not understand the rationale of exposure therapy because they believe they would be devalued for having requested an explanation.

Despite the crucial role DIBs play in the progression and treatment of mental disorders, there is no consensus of how broadly this construct should be defined,^1^ how it can be distinguished from other constructs (e.g., personality traits),^2^ and how exactly to determine the dysfunctionality of social cognitions^3^ [17, 18, 25–28]. While thoroughly answering these questions is beyond the scope of this paper, we focused on seeking trans-diagnostic, context-free instruments that could be utilized in clinical research. Unfortunately, we identified hardly any psychometric instrument capturing a broad range of DIBs as a trans-diagnostic marker regardless of a specific disorder (e.g., social anxiety [28]), population (e.g., children/adolescents [27]), social context (e.g., romantic relationships [17]), or different constructs (e.g., physiological activation [28]).

To the best of our knowledge, the Interpersonal Cognitive Distortions Scale (ICDS, [26]), which originally comprised an “interpersonal rejection”, “unrealistic relationship expectation”, and “interpersonal misperception” factor, is the only instrument that can be utilized to assess DIBs trans-diagnostically. However, to date, the ICDS has only been validated in a Turkish-speaking student sample using few clinical outcome measures. While the authors found substantial relationships with measures of irrational beliefs (*r* = .54), automatic thoughts (*r* = .53), and a tendency to interpersonal conflict (*r* = .53), inter-factor correlations (*r* = .07-.15) and internal consistencies (α = .43-.73) were rather low (model fit was acceptable to low; cf. [36]. Accordingly, it is still unclear to what extent the ICDS is suitable for predicting important clinical outcomes among different language-speakers and more clinical samples.

### Aims of the present study

To address these gaps, we aimed to validate the ICDS regarding its factor structure, internal consistency, and clinical validity using a heterogeneous sample from the German-speaking population that incorporated both clinical and non-clinical participants. Consistent with findings from Hamamci and Büyüköztürk [26], we expected moderate to large positive correlations between mean ICDS scores and indicators of similar constructs, like rejection sensitivity, interpersonal problems, and depressive expectations. Conversely, we expected moderate to large negative correlations with perceived social support, and interpersonal trust.

With respect to discriminant validity, we expected positive but lower correlations with perseverative negative thinking, and, conversely, lower negative correlations with general self-efficacy, and optimism.

In line with prior theorizing, we also hypothesized moderate to large positive correlations between mean ICDS scores and indicators of psychopathology and perceived stress, as well as moderate to large negative correlations with well-being.

Since chronic and treatment-resistant forms of mental disorders typically begin at a young age and research suggests that DIBs play a particularly important role in these forms [37, 38], we wondered whether an early onset of mental disorder would be associated with more DIBs and higher levels of psychopathology. This notion also fits well with findings from attachment research that DIBs primarily develop during childhood and adolescence, and then promote the development of mental problems during adulthood [39].

## Methods

The present study was approved by the local ethics committee of the University of Marburg (reference number: 2022-29k). All participants provided informed consent. The study was carried out in accordance with the ethical guidelines of the German Psychological Society. It was pre-registered at AsPredicted (https://aspredicted.org/4i22q.pdf).

### Participants

For recruitment, we used the online research platform Prolific (https://www.prolific.co), which is available for participants from most OECD countries. According to the Prolific Researcher Help Centre, participants are primarily recruited via word of mouth, including invitations by their social network on social media, in return for small cash incentives. We specified that participants had to be at least 18 years old and fluent in German. Participants were excluded if they discontinued their participation prematurely. To include both non-clinical and clinical participants in the final sample, we first applied the appropriate pre-filters in Prolific that asked participants about mental disorders (“Do you have - or have you had - a diagnosed, ongoing mental health illness/condition?”) and then drew participants from both pools. During the study course, participants were asked again about current or past mental disorders, based on which the final allocation was made. This resulted in a total sample of *N* = 216 participants, of whom *N* = 116 were classified as non-clinical and *N* = 100 as clinical individuals. As an incentive for participation, participants were paid 3.00£.

Data were collected between the 5^th^ and 15^th^ of August using the online tool Gorilla Experiment Builder (www.gorilla.sc). The questionnaire order was randomized for each participant.

### Instruments

#### Demographics

We assessed demographic variables like *gender, citizenship, migration history, relationship status, legal status, education, vocation, employment,* and *age* via self-report. We also assessed the age of *mental disorder onset* in years among our clinical participants*.*

#### DIBs

To assess DIBs, we had the English items of the ICDS [26] translated by a bilingual native speaker. Original items and their German translation are found in Appendix A. The ICDS comprises 19 statements related to dysfunctional interpersonal beliefs (e.g., “There are no real friends in this life”) that were rated on a 5-point Likert-type scale ranging from 1 (“I strongly disagree”) to 5 (“I strongly agree”). Higher composite scores indicate more DIBs. The internal consistency of the ICDS was acceptable (α = .76).

### Convergent validity

#### Rejection sensitivity

Similar to the assessment of DIBs, we assessed rejection sensitivity via a translated version of the Rejection Sensitivity Questionnaire for Adults (A-RSQ, [40]). Again, English items were translated with the help from a bilingual native speaker. The A-RSQ consists of nine items describing situations in which one approaches other people or asks them for something (e.g., “At a party, you notice someone on the other side of the room that you’d like to get to know, and you approach him or her to try to start a conversation”). Participants were instructed to imagine themselves in these situations and provide an individual concern-of-rejection rating (e.g., “How concerned or anxious would you be over whether or not the person would want to talk to you?”) as well as an expecting-acceptance rating (e.g., “I would expect that he/she would want to talk to me”), which were both rated on a 6-point Likert-type scale ranging from 1 (“very unconcerned”/”very unlikely”) to 6 (“very concerned”/”very likely”). Following the author’s recommendations, we computed a composite mean score by multiplying each concern-of-rejection rating with the inverse of the corresponding expectancy-of-acceptance rating, taking the mean of the resulting scores. Higher composite scores indicate higher rejection sensitivity. Internal consistency of the A-RSQ was good (α = .82).

#### Depressive expectations

Depressive expectations were measured by applying the German version of the Depressive Expectations Scale (DES, [41]). The DES consists of 25 expectations typical in people with depression (e.g., “When I ask someone for help, I will be rejected”). Each item is rated on a 5-point Likert-type scale ranging from 1 (“I disagree”) to 5 (“I agree”). Some items need to be inversed before computing the composite scores. Higher DES composite scores indicate more expectations that are typical of depression. The DES’s internal consistency in the present study was good (α = .88).

#### Interpersonal trust

The Social Trust Scale (STS, [42]) was administered to measure interpersonal trust. The STS consists of three questions about one’s beliefs about other people (e.g., “Do you think that most people would try to take advantage of you if they got the chance, or would they try to be fair?”), which were rated on a 11-point Likert-type scale ranging from 0 (e.g., “Most people would try to take advantage of me”) to 10 (e.g., “Most people would try to be fair”). Higher composite scores indicate higher interpersonal trust. Cronbach’s α indicated the ESS’s acceptable internal consistency (α = .77).

#### Interpersonal problems

To measure interpersonal problems, we employed the 32-item German translation of the Inventory of Interpersonal Problems (IIP-32, [43]; for the 64-item English version, see [44]). The IIP-32 comprises 32 items that describe difficulties in the relationship with others (e.g., “I argue with other people too much”). Participants were instructed to read through the list of items and consider whether any of the described difficulties applied to some aspect of their own relationship with significant others. Items were rated on a 5-point Likert-type scale ranging from 0 (“not at all”) to 4 (“extremely”), whereby higher composite scores indicate more interpersonal difficulties in relationships with others. The IIP-32’s internal consistency was good (α = .88).

#### Perceived social support

To assess perceived social support, we applied the German version of the Perceived Social Support Questionnaire (PSSQ-6, [45]). The PSSQ-6 encompasses six items mapping a wide range of social resources (e.g., “I know a very close person whose help I can always count on”). Items were rated on a 5-point Likert-type scale ranging from 1 (“not true at all”) to 5 (“very true”). Higher composite scores indicate higher perceived social support. The PSSQ-6’s internal consistency was good (α = .86).

### Discriminant validity

#### Self-efficacy

We measured general self-efficacy with the short scale to measure general self-efficacy beliefs (ASKU, [46]). The ASKU encompasses three items (e.g., “I can rely on my own abilities in difficult situations”) which were rated on a 5-point Likert-type scale ranging from 1 (“not true at all”) to 5 (“completely true”). Higher composite scores indicate stronger self-efficacy beliefs. Cronbach’s α indicated good internal consistency of the ASKU (α = .87).

#### Perseverative negative thinking

We applied the 15-item German version of the Perseverative Thinking Questionnaire (PTQ-15, [47]) as a measure of repetitive negative thinking. The PTQ-15 comprises 15 statements about one’s own thoughts or thought processes (e.g., “The same thoughts keep going through my mind again and again”), which were rated on a 5-point Likert-type scale ranging from 0 (“never”) to 4 (“almost always”). Higher composite scores indicate higher levels of perseverative negative thinking. Cronbach’s α indicated excellent internal consistency (α = .96).

#### Optimism

We assessed optimism using the 2-item German version of the Optimism-Pessimism-Scale (SOP-2, [48]), which consists of a definition of optimistic (“Optimists are people who look to the future with confidence and who mostly expect good things to happen”) and pessimistic people (“Pessimists are people who are full of doubt when they look to the future and who mostly expect bad things to happen”). Participants are instructed to answer a question on each definition (“How would you describe yourself? How optimistic/pessimistic are you in general?”) on a 7-point Likert-type scale ranging from 1 (“not optimistic at all”/”not pessimistic at all”) to 7 (“very optimistic”/”very pessimistic”). To obtain a composite score, the second item had to be reverse-scored. Higher composite scores indicate higher levels of optimism. The SOP-2’s internal consistency was good (α = .86).

### Clinical validity

#### Psychopathology

We used the short German version of the Brief Symptom Inventory (BSI-18, [49]; for the English version, see [50]) to derive indices of psychopathology. The BSI-18 consist of three subscales (“Somatization”, “Depression”, “Anxiety”) à six symptoms (e.g., “feeling lonely”) which were rated on a 5-point Likert-type scale ranging from 0 (“not at all”) to 4 (“extremely”). Participants should indicate how much they had suffered from the listed symptoms in the past seven days. Higher composite scores indicate higher levels of psychopathology. Cronbach’s α indicated excellent internal consistency of the BSI-18 (α = .92).

#### Perceived stress

We assessed perceived stress using the German items of the short version of the Perceived Stress Scale (PSS-4, [51]; for the English version, see [52]). The PSS-4 consists of four items (e.g., “In the last month, how often have you felt that things were going your way”) rated on a 5-point Likert-type scale ranging from 0 (“never”) to 4 (“very often”). Item two and three needed to be inversed. Higher composite scores indicate more perceived social stress. Cronbach’s α indicated acceptable internal consistency (α = .79).

#### Well-being

The German 5-item version of the World Health Organization-Five Well-Being Index (WHO-5, [53]; for the English version, see [54]) was used to measure well-being. Items (e.g., "I have felt cheerful and in good spirits") referred to "the past two weeks" and were rated on a 6-point Likert scale ranging from 0 ("at no time") to 5 ("all of the time"). Higher composite scores indicate higher levels of well-being. Cronbach's α indicated excellent internal consistency (α = .91).

#### Data preparation and statistical analyses

Eight participants (with standardized mean scores and variances computed over all scales that where *z* > 3) were excluded from our analyses because they showed suspicious response patterns. All analyses were run using Jamovi [55] or JASP [56] in conjunction with the *R*-packages [57] *psych* [58] and *lavaan* [59].

We chose to conduct EFA rather than principal component analysis (PCA) because we wanted to identify a latent factor structure.^4^ Although we originally considered analyzing clinical and non-clinical participants separately, we ultimately decided to base our main analyses on the pooled sample (*N* = 208) to improve the power of our EFA. Both Bartlett’s test (χ^2^ (171) = 1451.52, *p* < .001) and the Kaiser–Meyer–Olkin test (MSA = 0.75) indicated that our data were suitable for conducting EFA. Because Mardia’s test indicated a violation of multivariate normality (all *ps* < .001), we decided to base our EFA on the polychoric correlation matrix and used principal axis factoring with promax rotation for factor extraction [60]. Following the recommendations of Watkins (2018), we used parallel analysis to determine the number of factors. Pattern coefficients above .40 were considered salient. Item analyses were conducted in line with the recommendations of Moosbrugger and Kelava [61].

To assess convergent (rejection sensitivity, depressive expectations, interpersonal trust, interpersonal problems, perceived social support), discriminant (self-efficacy, perseverative negative thinking, optimism), and clinical (psychopathology, perceived stress, well-being) validity of the ICDS, we conducted bivariate correlational analyses using Spearman’s ρ. Moreover, we conducted an exploratory mediation analysis with disorder onset in years as predictor variable, a mean ICDS subscale score as mediator variable, and the mean BSI-18 score as dependent variable to test whether the effect of mental disorder onset on levels of psychopathology was mediated by higher levels of DIBs. We used bootstrapping (2000 samples) to account for non-normality. Note that the mediation analysis was based on *N* = 87 participants due to missing values across the clinical participants.

## Results

Our data can be accessed at https://data.uni-marburg.de/handle/dataumr/230.3.

### Sample characteristics

Sample characteristics are found in Table 1. Gender proportions, mean age and education level were quite similar when comparing non-clinical and clinical participants descriptively. As expected, clinical participants had higher mean scores on indicators of DIBs, rejection sensitivity, psychopathology, depressive expectations, interpersonal problems, perceived stress, and perseverative negative thinking. Clinical participants also reported higher levels of perceived social support, which may seem counterintuitive at first, but may simply be explained by their greater need of (and, in turn, receipt of) social support. Conversely, non-clinical participants had higher mean scores on indicators of self-efficacy, interpersonal trust, optimism, and well-being. Somewhat surprisingly, the clinical participants reported slightly lower scores on the ICDS’s “misperception” subscale, which we discuss below in more detail.

**Table 1:** Sample characteristics

	**“Non-clinical” participants** (*N* = 114)	**“Clinical” participants** (*N* = 94)	**All participants** (*N* = 208)
**Gender**			
male, *N* (%)	61 (53.51)	40 (42.55)	101 (48.56)
female, *N* (%)	53 (46.49)	50 (53.19)	103 (49.52)
diverse, *N* (%)	0 (0.00)	4 (4.26)	4 (1.92)
**Citizenship**			
German, *N* (%)	41 (35.96)	58 (61.70)	99 (47.60)
other, *N* (%)	73 (64.04)	36 (38.30)	109 (52.40)
**Migration history**			
immigrated, *N* (%)	16 (14.04)	9 (9.57)	25 (12.02)
not immigrated, *N* (%)	98 (85.96)	85 (90.43)	183 (87.98)
**Relationship status**			
single, *N* (%)	58 (50.88)	46 (48.94)	104 (50.00)
partner, *N* (%)	56 (49.12)	48 (51.06)	104 (50.00)
**Legal status**			
unmarried, *N (%)*	74 (64.91)	73 (77.66)	147 (70.67)
married, *N* (%)	28 (24.56)	15 (15.96)	43 (23.08)
registered partnership, *N* (%)	6 (5.26)	1 (1.06	7 (3.37)
divorced, *N* (%)	5 (4.39)	4 (4.26)	9 (4.33)
widowed, *N* (%)	1 (0.88)	1 (1.06)	2 (0.96)
**Education**			
high school diploma, *N* (%)	90 (78.95)	71 (75.53)	161 (77.40)
secondary school diploma, *N* (%)	24 (21.05)	22 (23.40)	46 (22.12)
no school diploma, *N* (%)	0 (0.00)	1 (1.06)	1 (0.48)
**Vocation**			
PhD, *N* (%)	6 (5.26)	2 (2.13)	8 (3.85)
master’s degree, *N* (%)	26 (22.81)	21 (2.23)	47 (22.60)
bachelor’s degree, *N* (%)	43 (37.72)	17 (18.09)	60 (28.85)
professional vocation, *N* (%)	23 (20.18)	17 (18.09)	40 (19.23)
no vocation, *N* (%)	16 (14.04)	37 (39.36)	53 (25.48)
**Employment**			
full-time, *N* (%)	63 (55.26)	28 (29.79)	91 (43.75)
part-time, *N* (%)	27 (23.68)	36 (38.30)	63 (30.29)
not employed, *N* (%)	24 (21.05)	30 (31.91)	54 (25.96)
**Age**, *M (SD)*	33.80 (13.16)	28.57 (8.93)	31.44 (11.71)
**ASKU**, *M (SD)*	3.93 (0.79)	3.46 (0.87)	3.72 (0.86)
**A-RSQ**, *M (SD)*	9.73 (3.89)	10.98 (4.63)	10.30 (4.28)
**BSI-18**^**a**^			
Overall	0.88 (0.70)	1.41 (0.80)	1.12 (0.79)
somatization, *M (SD)*	0.63 (0.68)	0.97 (0.84)	0.79 (0.77)
depression, *M (SD)*	1.06 (0.91)	1.76 (1.08)	1.37 (1.05)
anxiety, *M (SD)*	0.94 (0.75)	1.49 (0.88)	1.19 (0.85)
**DES**^**a**^, *M (SD)*	2.37 (0.56)	2.62 (0.57)	2.48 (0.58)
**STS**^**a**^, *M (SD)*	5.62 (1.76)	5.21 (1.96)	5.43 (1.86)
**ICDS**, *M (SD)*	2.75 (0.48)	2.93 (0.46)	2.83 (0.48)
“insecurity”, *M (SD)*	2.18 (0.85)	2.60 (0.86)	2.37 (0.88)
“dependency”, *M* (SD)	2.63 (0.79)	2.72 (0.85)	2.67 (0.81)
“demandingness”, *M (SD)*	3.68 (0.77)	4.04 (0.72)	3.84 (0.77)
“misperception”, *M (SD)*	2.78 (0.84)	2.69 (0.79)	2.74 (0.82)
“distrust”, *M (SD)*	2.86 (0.69)	2.90 (0.74)	2.87 (0.71)
**IIP-32**, *M (SD)*	1.58 (0.56)	1.72 (0.56)	1.64 (0.56)
**PSS**^**a**^, *M (SD)*	1.68 (0.86)	2.16 (0.87)	1.89 (0.89)
**PSSQ**^**a**^, *M (SD)*	3.51 (0.98)	3.55 (0.86)	3.53 (0.93)
**PTQ-15**^**a**^, *M (SD)*	1.89 (0.90)	2.50 (0.95)	2.17 (0.97)
**SOP-2**, *M (SD)*	4.52 (1.41)	3.65 (1.41)	4.13 (1.47)
**WHO-5**^**a**^, *M (SD)*	2.98 (1.13)	2.37 (1.11)	2.70 (1.16)

*N* Sample size, *M* Mean, *SD* Standard deviation

^a^Please note that we report mean composite scores instead of mean sum scores here

### Exploratory factor analysis and item analyses

Parallel analyses in conjunction with the inspection of the scree plot revealed a five-factor solution accounting for 50.4% of the variance. Model fit indices indicated poor to acceptable model fit (Appendix B). Table 2 shows the factor matrix and important item characteristics of the German translation of the ICDS. There were no cross-loadings above .40. The rather low (and in some cases even slightly negative) inter-factor correlations (Table 3) indicated that the factors were largely independent of each other (for inter-item correlations, see Appendix C). Therefore, we decided to conduct item analyses at both the total scale and subscale levels based on item-rest correlations, item difficulties, internal consistencies, and item content. Following the recommendations of Moosbrugger and Kelava [62], we decided to discard none of the items.

**Table 2:** Factor matrix of the translated version of the ICDS

Original items^a^		Factor loadings					Item characteristics						
		i	ii	iii	iv	v	*M*	*SD*	*Sk*	*K*	*U*	*r*_rest_	*D*
1	Being intimate with people usually creates problems	.60					2.33	1.10	0.49	-0.67	.49	.37	33.25
2	People do not understand me	.75					2.71	1.16	0.12	-0.89	.41	.44	42.75
3	I believe that I will be rejected if I reveal my feelings and thoughts to people around me	.82					2.61	1.27	0.36	-0.95	.32	.49	40.25
4	There are no real friends in this life	.53					1.78	1.10	1.24	0.50	.34	.40	19.50
5	I want people that [sic] I am in contact with to share their feelings and thoughts with me			.62			3.95	1.03	-0.84	0.04	.58	.12	73.75
6	I understand from someone’s eyes what kind of a person they are				.71		2.86	1.12	-0.13	-0.84	.50	.19	46.50
7	I feel what they think even if people do not show it				.85		3.19	1.06	-0.42	-0.56	.30	.28	54.75
8	Other people should know what I think even if I do not reveal my thoughts				.49		2.17	0.99	0.54	-0.43	.60	.32	29.25
9	To feel good, other people’s thoughts and feelings about me should be positive		.68				2.91	1.17	-0.19	-0.96	.45	.40	47.75
10	People do not keep their promises					.63	2.77	1.06	0.20	-0.52	.36	.37	44.25
11	I should always belong to a social group		.68				2.57	1.11	0.30	-0.80	.60	.27	39.25
12	I believe that people do not accept me when I am in a social environment	.81					2.42	1.25	0.58	-0.74	.41	.39	38.50
13	It is beneficial to be alert to people around us					.46	2.96	1.11	-0.03	-0.77	.54	.43	49.00
14	I should be tolerant of others in order not to offend them			.74			3.80	0.97	-0.56	-0.20	.47	.17	70.00
15	I should behave as others want me to behave in order to make them happy		.45				2.74	1.24	0.28	-0.92	.69	.37	43.50
16	I always want somebody to be around me		.53				2.46	1.21	0.32	-0.95	.68	.30	36.50
17	I always want people to show understanding to me			.59			3.77	0.96	-0.76	0.40	.48	.29	69.25
18	People should meet each other’s expectations in relationships					.48	3.32	0.99	-0.34	-0.25	0.60	.32	58.00
19	It is always useful to keep superficial [*sic*] our relationships with others					.44	2.45	1.05	0.20	-0.68	0.56	.27	36.25

*Notes*. *N* Sample size, i “Insecurity” subscale, ii “Dependency” subscale, iii “Demandingness” subscale, iv “Misperception” subscale, v “Distrust” subscale, *M* Mean, *SD* Standard deviation, *Sk* Skewness, *K* Kurtosis, *U* Uniqueness, *r*_rest_ Item-rest correlation, *D* Item difficulty

^a^Original items from Hamamci and Büyüköztürk [26]. The corresponding translations can be found in Appendix A

**Table 3:** Inter-factor correlations

	**Insecurity**	**Dependency**	**Demandingness**	**Misperception**	**Distrust**
Insecurity	-	.14	-.15	.14	.36
Dependency	.14	-	.33	.28	.13
Demandingness	-.15	.33	-	-.01	-.26
Misperception	.14	.28	-.01	-	.22
Distrust	.36	.13	-.26	.22	-

We report Pearson's *r* here

The internal consistency of the ICDS was acceptable at the total scale level (α = .76), but rather poor at the subscale level (“dependency”: α = .63, “demandingness”: α = .67, “misperception”: α = .66, “distrust”: α = .61), although the “insecurity” subscale had good internal consistency (α = .80).

### Validity analyses

#### Convergent validity

Regarding convergent validity, bivariate correlational analyses revealed substantial correlations between the ICDS and indicators of rejection sensitivity (A-RSQ), depressive expectations (DES), interpersonal trust (STS), interpersonal problems (IIP-32), and perceived social support (PSSQ-6) at the total scale level following the predicted direction (Table 4). While most of these correlations were of expected magnitude, the correlations with indicators of rejection sensitivity (A-RSQ) and perceived social support (PSSQ-6) were slightly lower than expected.

**Table 4:** Validity analyses

	I	i	ii	iii	iv	v	II	III	IV	V	VI	VII	VIII	IX	X	XI	XII
I. ICDS	-																
i. Insecurity	**.69**	-															
ii. Dependency	**.63**	**.15**	-														
iii. Demandingness	**.30**	-.09	**.36**	-													
iv. Misperception	**.46**	.09	**.19**	.10	-												
v. Distrust	**.68**	**.49**	**.22**	-.07	**.21**	-											
II. A-RSQ^a^	**.27**	**.55**	-.05	-.13	-.10	**.25**	-										
III. DES^a^	**.57**	**.78**	**.14**	-.12	.07	**.42**	**.62**	-									
IV. STS^a^	**-.35**	**-.50**	.10	**.14**	-.07	**-.52**	**-.35**	**-.44**	-								
V. IIP-32^a^	**.59**	**.60**	**.33**	.09	.04	**.39**	**.45**	**.60**	**-.26**	-							
VI. PSSQ-6^a^	**-.29**	**-.59**	.07	**.33**	.04	**-.32**	**-.56**	**-.61**	**.35**	**-.37**	-						
VII. ASKU^b^	**-.27**	**-.36**	-.13	-.10	.11	**-.18**	**-.35**	**-.48**	**.23**	**-.38**	**.30**	-					
VIII. PTQ-15^b^	**.52**	**.50**	**.21**	**.30**	.13	**.29**	**.37**	**.60**	**-.30**	**.49**	**-.20**	**-.47**	-				
IX. SOP-2^b^	**-.38**	**-.53**	.07	-.07	-.03	**-.35**	**-.39**	**-.57**	**.53**	**-.29**	**.41**	**.46**	**-.50**	-			
X. BSI-18^c^	**.44**	**.55**	.11	.05	.11	**.29**	**.43**	**.68**	**-.36**	**.46**	**-.41**	**-.47**	**.67**	**-.52**	-		
XI. PSS-4^c^	**.36**	**.50**	.02	.06	-.04	**.25**	**.45**	**.63**	**-.34**	**.41**	**-.45**	**-.57**	**.62**	**-.57**	**.66**	-	
XII. WHO-5^c^	**-.21**	**-.47**	.03	.01	**.14**	**-.14**	**-.42**	**-.56**	**.27**	**-.20**	**.52**	**.36**	**-.43**	**.54**	**-.53**	**-.61**	-

Bold numbers indicate significant values (*p* < .05)

^a^Indicator of convergent validity

^b^Indicator of discriminant validity

^c^Indicator of clinical validity

This correlational pattern could be replicated (and even sharpened) at the subscale level when investigating the corresponding correlations using the “insecurity” or the “distrust” subscale (Table 4). However, the correlational pattern was less clear cut concerning the other three subscales and only partially followed the hypothesized direction. Contrary to our hypotheses, the “misperception” subscale revealed no substantial correlation with any relevant indicator of convergent validity at all. Moreover, while the “dependency” subscale showed lower and sometimes even non-significant associations with indicators of convergent validity, the “demandingness” subscale revealed two correlations with indicators of interpersonal trust (STS) and perceived social support (PSSQ-6) that followed an unexpected direction. We discuss this finding in more detail below.

#### Discriminant validity

Concerning discriminant validity, bivariate correlations followed the predicted direction at the total scale level but were larger than expected in the case of perseverative negative thinking (PTQ-15) and optimism (SOP-2).

While this pattern was largely replicated at the subscale level when the corresponding correlations were examined using the “insecurity”, the “distrust”, or the “demandingness” subscales (sometimes with other indicators of discriminant validity, Table 2), no substantial or low (but correctly directed) correlations were found when the other subscales were examined.

#### Clinical validity

With respect to clinical validity, our analyses revealed substantial correlations with indicators of psychopathology (BDI-18), perceived stress (PSS-4), and well-being (WHO-5) at the total scale level that followed the predicted direction and were mostly of expected magnitude. Only the correlation with well-being was somewhat lower than expected.

At the subscale level, only the “insecurity”, “misperception”, and “distrust” subscales revealed substantial correlations with indicators of clinical validity. While almost all of these correlations followed the hypothesized direction and, in case of the “insecurity” subscale, were of expected magnitude, the “misperception” subscale showed a substantial positive correlation with well-being (WHO-5), which was not expected and is discussed in more detail below.

Please note that although the “dependency” and “demandingness” subscales revealed no substantial associations with the clinical validity indicators selected here, they did show substantial associations with other clinically meaningful variables, such as interpersonal problems (IIP-32) or perseverative negative thinking (PTQ-15), addressed already in the other validity analyses.

Overall, our predictions regarding convergent, discriminant, and clinical validity were mostly supported at the total scale level, with observed correlations always following the hypothesized direction but not always reaching the expected magnitude. At the subscale level, our results were not as consistent and less clear, so we found our hypotheses only partially supported here.

#### Exploratory mediation analysis

As the correlations between the factors were negative in some cases, we decided to conduct our exploratory mediation analysis at the subscale level, using the mean of the “insecurity” subscale as the mediator variable, since that was the only subscale with good internal consistency. This analysis revealed a substantial total effect of *disorder onset* on *psychopathology* (BSI-18; *B* = -0.03 *SE* = 0.01, *z* = -2.58, *p* = .010, 95%-CI = [-0.05; -0.01]) and a substantial indirect effect (*B* = -0.01, *SE* = 0.01, *z* = -2.43, *p* = .015, 95%-CI = [-0.03; -0.01]). The direct effect failed to reach significance (*B* = -0.02, *SE* = 0.01, *z* = -0.143, *p* = .154, 95%-CI = [-0.04; 0.01]). All path coefficients followed the expected direction (*disorder onset *on *DIBs*: *b* = -0.04, *SE* = 0.01, *z* = -3.00 *p* = .003, 95%-CI = [-0.06; -0.02]; *DIBs* on *psychopathology*: *b* = 0.37, *SE* = 0.09, *z* = 4.11, *p* < .001, 95%-CI = [0.19; 0.52]; *disorder onset* on *psychopathology*: *b* = -0.02, *SE* = 0.01, *z* = -1.43*, p* = .154, 95%-CI = [-0.04; 0.01]). The mediation model accounted for 22.2% of the variance in *psychopathology* (BSI-18) and for 9.4% of the variance in *DIBs* (i.e., the mean “insecurity” subscale score).

## Discussion

In the present study, we psychometrically tested and validated a German translation of the ICDS within a heterogeneous, German-speaking sample along with measures of convergent (rejection sensitivity, depressive expectations, interpersonal trust, interpersonal problems, perceived social support), discriminant (self-efficacy, perseverative negative thinking, optimism), and clinical validity (psychopathology, perceived stress, well-being).

### Factor structure

The results of our EFA indicate a five-factor structure of the ICDS’s German version, which partly corresponds to the original scale’s structure concerning item-factor allocation, but also shows considerable discrepancies (cf. [26], Table 2). While some of these discrepancies are attributable to translation aspects and cultural differences, and to differences in the statistical approach, we mainly attribute them to our sample’s greater heterogeneity in demographic (e.g., age and education) and clinical variables (e.g., psychopathology levels). This seems plausible, as heterogeneous samples naturally cover a broader spectrum of the construct space. In addition, we believe that the more differentiated factorization also better reflects the item contents. Considering the comparatively high factor loadings and comparable internal consistencies along with the more parsimonious model assumptions, and the EFA’s comparable model fit, we conclude that the original publication’s factor structure should not be applied to the German version when investigating heterogeneous or more clinical populations.^5^

Similar to the original scale, however, low (and in some cases even negative) inter-factor correlations indicate that the ICDS German version should be interpreted primarily at the subscale level. This finding also suggests that DIBs constitute a multidimensional construct, where high scores on one factor may exclude (or at least be independent of) high scores on the other.^6^

Considering the rather weak internal consistencies, the low eigenvalues of some factors (Appendix D), and the still suboptimal model fit (Appendix B), all the subscales need further improvement. This could probably be achieved in future research by adding more items to the corresponding subscales (to increase the construct validity) and by adding further facets of DIBs not covered by the ICDS so far. These could include, for example, beliefs about being a burden to other people [63], being threatened or exploited by others [64], or finding no pleasure in relationships with other people [65].

### Validity

#### Convergent validity

Our findings suggest acceptable convergent validity of the German version of the ICDS at the total scale level. The correlations’ direction and magnitude were largely consistent with our hypotheses. However, since we are best advised to interpret our findings at the subscale level, these results should be discussed with some differentiation. For example, our findings from the “insecurity” subscale indicate good convergent validity and are consistent with a large body of literature suggesting a close relationship between DIBs related to social insecurity and measures of convergent validity (for empirical studies, see [6, 16, 41, 66, 67]; for research-syntheses, see e.g., [22, 68, 69]). The same applies to the “distrust” subscale, which also revealed the hypothesized correlational pattern - albeit with somewhat lower correlations than expected. While the latter could be attributed to the subscale’s low internal consistency and suboptimal construct validity, the pattern of findings itself is consistent with the literature suggesting hypothesized associations with measure of convergent validity [70–73].

In contrast, our findings regarding the “dependency” and “demandingness” subscales only partially followed the predicted directions and were, overall, lower than expected.^7^ While these associations are again probably attributable to poor internal consistencies and a lack of construct validity, some associations found seem to be consistent with the literature [77].

Finally, the “misperception” subscale exhibited no substantial relationship with any of the aforementioned constructs at all. Although there were problems with internal consistency and construct validity with this scale too, a closer look at the item content suggests another explanation. It is possible that the predicted correlations occur only in very extreme samples (e.g., psychotic or forensic individuals), where the items may take on a different meaning and reflect serious misperceptions of interpersonal signals [18].

In summary, convergent validity at the subscale level was only partially given, which can be attributed to the psychometric weaknesses of some subscales (low internal consistency, lack of construct validity), but also to the multidimensionality of the DIB construct (see the section on factor structure). In addition to the already suggested improvement and extension of the ICDS, future research should conduct subscale-specific validity analyses and select validity measures that are also subscale-specific.

#### Discriminant validity

At the overall scale level, our findings suggest good discriminant validity concerning the indicator of general self-efficacy, which is in line with our hypotheses. However, we found suboptimal discriminant validity regarding our indicators of perseverative negative thinking and dispositional optimism. The tendency for negative perseverative thinking appears to be more closely tied to the occurrence of DIBs than expected, suggesting that metacognitive processes such as rumination can substantially increase the occurrence of negative cognitions in general [78, 79]. The unexpected moderate correlation with our indicator of dispositional optimism may be due to the fact that the negative social beliefs indicated by the ICDS may actually reflect a critical outcome of low dispositional optimism [80].

At the subscale level, we can assume good discriminant validity for all subscales, but with limitations for the “insecurity” and “distrust” subscales, which showed deviations from our hypotheses comparable to those found for the overall scale (probably for reasons we discussed above).

It should be noted that our analyses on discriminant validity should be replicated at the subscale level in future studies (e.g., after the expansion of ICDS) to ensure that the overall good discriminant validity is not solely the result of rather weak psychometric properties.

#### Clinical validity

Substantial correlations between the ICDS total score and indicators of clinical validity demonstrated acceptable clinical validity of the ICDS’s German version, although the correlation with the WHO-5 was somewhat lower than expected. We attribute the latter to the fact that we found opposing correlations at the subscale level (Table 4). For example, while the “insecurity” subscale showed a moderate negative correlation with well-being in line with our hypotheses, we observed a slightly positive correlation with the “misperception” subscale. As mentioned above, this differential effect, which seems counterintuitive at first glance, may be explained by the fact that for the “misperception” subscale, substantial correlations with clinical variables occur only at very extreme levels and that positive outcomes may dominate at less extreme levels (cf. findings on narcissism [81]).

At the subscale level, clinical validity seems to be given especially for the “insecurity” subscale and partly also for the “distrust” subscale. In accordance with the literature, the expected correlations with indicators of psychopathology, perceived stress, and well-being were found, although they were somewhat weaker than expected with the “distrust” subscale (for empirical studies, see [82–86]; for reviews, see [69, 87]). While the latter may again be related to weak internal consistency and questionable construct validity,^8^ almost none of the other subscales revealed any substantial associations with any of the clinical variables.^9^

#### Do DIBs mediate the relationship between disorder onset and psychopathology?

Consistent with previous research, we found that DIBs (as measured by the mean “insecurity” subscale score) mediate the negative relationship between mental disorder onset and psychopathology, thus suggesting their relevance in the development and maintenance of mental disorders, including their particular role in early-onset mental disorders [93]. The reader should note, however, that we can draw no causal conclusions based on the cross-sectional data analyzed here. Overall, 22.2% of the variance in psychopathology (as indicated by the BSI-18) and 9.4% of the variance in DIBs could be explained by our mediation model.

#### Limitations and future directions

Although our study has several strengths, such as incorporating clinical and nonclinical participants, there are limitations that warrant discussion.

First, please note that our hypotheses on the validity of the ICDS's German version initially focused on the ICDS’s total score, so that we selected all validity measures with regard to the total scale. However, since the factor analysis demonstrated rather low, partly even negative, inter-factor correlations, the question arises whether the selected measures are still suitable to evaluate convergent, discriminant, and clinical validity, at the subscale level. Because DIBs appear to be a multidimensional construct, future research should engage in unique validity analyses for each subscale. In this context, we also believe that the clinical utility of the “dependency”, “demandingness”, and “misperception” subscales was insufficiently clarified in our study because of the respective scales’ lack of internal consistency and construct validity. While our findings tend to argue against the clinical utility of these scales, we recommend adding additional items to the subscales in future studies to achieve better construct validity. Furthermore, it would be worthwhile validating the ICDS in more extreme (e.g., psychotic or forensic) samples, since some items and scales may reveal stronger clinical validity here.

Second, while reviewing the literature, we noticed that important types of DIBs are missing or not sufficiently captured in the ICDS’s current version. These include, for example, beliefs about being a burden to other people [63], being threatened or exploited by others [64], or finding no pleasure in relationships with other people [65]. Future studies should investigate the extent to which the ICDS should be extended at the subscale and item levels regarding these facets.

Third, our study’s cross-sectional design enabled us to draw conclusions about neither the temporal stability of the ICDS and its subscales nor the temporal precedence of DIBs and clinical outcomes. Future investigations of whether DIBs precede or follow psychopathological developments seem eminently worthwhile to us, as this would shed light on whether DIBs should be considered a risk factor for, or rather a consequence of mental disorders [6].

Finally, our study does not provide any evidence of the extent to which people with different mental disorders may reveal diverse, probably opposing, response patterns on the ICDS and its subscales. However, knowledge about which clusters of DIBs coincide with various groups of mental disorders would be of great interest for both clinical practice and etiological research. This becomes clear in light of the fact that individuals with different mental disorders are also likely to differ markedly in the content of their DIBs [94].

## Conclusion

The present study evaluated a German translation of the ICDS with regard to its factor structure, internal consistency, and validity, investigating a heterogeneous, German-speaking sample that incorporated clinical and non-clinical participants. Our results indicated that the factor structure of the ICDS's German version differs substantially from the structure proposed in the original publication, although some item-factor allocations could be replicated [26]. Moreover, while especially the “insecurity” subscale revealed acceptable psychometric properties, we suspect that the other subscales’ low validity may be at least partially attributable to low internal consistencies and a lack of construct validity. Therefore, future studies should improve the “dependency”, “demandingness”, “misperception”, and “distrust subscales” by adding more items to increase the subscales’ construct validity. In addition, as outlined above, researchers should expand the ICDS to include other classes of DIBs and examine its validity in samples presenting more extreme forms of DIBs (e.g., forensic or psychotic samples). In conclusion, due to negative inter-factor correlations and clinical considerations, we strongly recommend that DIBs (measured with the ICDS’s German version) be considered as a multidimensional construct. Future validity analyses and interpretations should be conducted exclusively at the subscale level.

### Supplementary Information

**Additional file 1:** **Appendix A. **Original items of the ICDS with the German translations.

**Additional file 2: Appendix B.** Model fit indices of the EFA that was based on the pooled sample.

**Additional file 3:** **Appendix C.** Inter-item correlations of the ICDS.

**Additional file 4:** **Appendix D.** Scree plot from the EFA that was based on the pooled sample.

**Additional file 5:** **Appendix E.** Model fit indices of the CFA using the three-factor solution from Hamamci & Büyüköztürk (2004) on the pooled sample.

## Data Availability

The datasets generated and/or analyzed during the current study are available in the research data repository of the Philipps-University of Marburg, https://data.uni-marburg.de/handle/dataumr/230.3.

## Footnotes

1 For the purposes of this paper, we define DIBs as explicitly or implicitly represented cognitions about other people or current and future interactions and relationship with those people that are likely to adversely affect the belief-holder's mental health or survival.

2 According to the literature, explicitly represented cognitions such as “beliefs” or “expectations” concerning the relationship with others appear to fall under this construct [17, 18, 25–28]. However, it is largely unclear to what extent other constructs from different research areas, such as relational schemas [29], social expectations [30, 31], attributions within close relationships [32], attitudes and implicitly represented social beliefs [33], or relatively stable dispositions [34], should not also be subsumed under this construct. What seems clear is that both implicit or explicit wishes and hopes (e.g., “I would wish for a better relationship with other people”) that refer to desired or hoped-for interpersonal outcomes rather than “probability-driven assessment[s] of the most likely outcome” do not fall under this construct ([35], p. 347). This also applies to past-related interpersonal cognitions (e.g., “People never liked me”), which may implicitly reflect current beliefs but do not necessarily have to.

3 We suggest assessing a social cognition’s dysfunctionality by the extent to which it negatively affects the mental health or survival of the belief holder.

4 Moreover, we decided against conducting a CFA in our main analyses as the factor structure proposed by Hamamci and Büyüköztürk [26] was obtained using PCA, which is not recommended when attempting to identify latent constructs responsible for variations in the measured variables [60]. Another reason was that Hamamci’s and Büyüköztürk’s [26] findings relied on a sample very different from ours in terms of language, demographics, and clinical characteristics. Nevertheless, those interested in the degree to which Hamamci’s and Büyüköztürk’s [26] factor structure fits the data of our study are referred to Appendix D.

5 Please note that, despite the above arguments, some eigenvalues turned out to be quite low (Appendix D). Therefore, we strongly recommend to replicate our findings in future studies, possibly using an improved version of the ICDS*.*

6 From the practitioner’s point of view, this makes sense, as individuals with different clinical characteristics may score very differently (and even opposed to each other) on the respective subscales [22]. For example, an insecure social phobic patient could conceivably score higher on the “insecurity” and “dependency” subscales than a narcissistic patient who would probably score higher on the “demandingness” and “misperception” subscales. In other words, scoring high on some subscales of the ICDS’s German version could be mutually exclusive and potentially associated with other clinical outcomes.

7 The absence of significant correlations between rejection sensitivity, interpersonal trust, and perceived social support for the “dependency” subscale may be due to the fact that the pursuit of social affiliation does not always arise from a deficient motivation [74, 75]. The “dependency” subscale revealed only consistently weak positive correlations with the “insecurity” subscale, which captured aspects of rejection sensitivity and interpersonal trust. In this context, Lavigne and colleagues [75] suggest that the extent to which striving for social affiliation is associated with lower or higher interpersonal functioning depends on the  underlying motivation (“growth orientation” vs. “deficit reduction”). The non-significant correlations between the “demandingness” subscale and rejection sensitivity, depressive expectations, or interpersonal problems, on the other hand suggest a somewhat different explanation. Individuals who are demanding in relationships may often reveal rather low social insecurity, harbor more optimistic expectations and normal levels of interpersonal trust because they may be more successful in asserting and actively caring for their needs [76]. This would also explain why the “demandingness” subscale correlates negatively with the “insecurity” and “distrust” subscales. Nevertheless, at least for high levels of social demandingness, one would expect a substantial correlation with interpersonal problems [18], which our data fails to demonstrate. However, it is possible that this correlation is only revealed in more extreme (e.g., forensic) samples.

8 With regard to the “demandingness” subscale, for example, there would be other aspects inadequately captured by the eventual items, such as the belief that others should bear more of the stress you are experiencing (e.g., [88]) or that they should take more responsibility for you [89].

9 One exception that has been discussed is the substantial association between the “misperception” subscale and the WHO-5. Apart from the subscales’ aforementioned psychometric weaknesses, we believe that other causes, some of which are discussed above, may also play a role in this result. These include the idea that some of the subscales may only be associated with clinical variables in more extreme (e.g. forensic or psychotic) samples [90–92] and may even be initially associated with more positive clinical outcomes in less extreme samples (cf. e.g., with findings on narcissism [81]).
