# Supplementary material for: Measuring dysfunctional interpersonal beliefs: validation of the Interpersonal Cognitive Distortions Scale among a heterogeneous German-speaking sample
Source: BMC Psychiatry. 2023 Sep 27;23:702. doi: 10.1186/s12888-023-05155-3 (PMC10523705; doi:10.1186/s12888-023-05155-3)
Supplement: Supplementary file 1 — Additional file 1: Appendix A. Original items of the ICDS with the German translations. [file 12888_2023_5155_MOESM1_ESM.docx]

| **Appendix A.** Original items of the ICDS with the German translations. | | | |
| --- | --- | --- | --- |
| **Original item** | | **German translation** | |
| 1. | Being intimate with people usually creates problems. | 1. | Mit anderen Menschen vertraut zu sein, schafft in der Regel Probleme. |
| 2. | People do not understand me. | 2. | Die Menschen verstehen mich nicht. |
| 3. | I believe that I will be rejected if I reveal my feelings and thoughts to people around me. | 3. | Ich glaube, dass ich zurückgewiesen werde, wenn ich den Menschen um mich herum offen meine Gefühle und Gedanken zeige. |
| 4. | There are no real friends in this life. | 4. | Es gibt keine echten Freunde im Leben. |
| 5. | I want people that I am in contact with to share their feelings and thoughts with me. | 5. | Ich möchte, dass die Menschen, mit denen ich in Kontakt stehe, ihre Gefühle und Gedanken mit mir teilen. |
| 6. | I understand from someone’s eyes what kind of a person they are. | 6. | Ich kann jemandem von den Augen ablesen, was für ein Mensch er ist. |
| 7. | I feel what they think even if people do not show it. | 7. | Ich fühle, was Leute denken, auch wenn sie es nicht zeigen. |
| 8. | Other people should know what I think even if I do not reveal my thoughts. | 8. | Andere Menschen sollten wissen, was ich denke, auch wenn ich meine Gedanken nicht offen zeige. |
| 9. | To feel good, other people’s thoughts and feelings about me should be positive. | 9. | Damit ich mich gut fühlen kann, sollten andere Menschen positive Gedanken und Gefühle mir gegenüber haben. |
| 10. | People do not keep their promises. | 10. | Menschen halten ihre Versprechen nicht. |
| 11. | I should always belong to a social group. | 11. | Ich sollte immer einer sozialen Gruppe angehören. |
| 12. | I believe that people do not accept me when I am in a social environment. | 12. | Wenn ich in einer sozialen Umgebung bin, glaube ich, dass mich die Menschen nicht akzeptieren. |
| 13. | It is beneficial to be alert to people around us. | 13. | Es ist von Vorteil, vor den Menschen um uns herum auf der Hut zu sein. |
| 14. | I should be tolerant of others in order not to offend them. | 14. | Ich sollte anderen Menschen gegenüber tolerant sein, um sie nicht zu verletzten. |
| 15. | I should behave as others want me to behave in order to make them happy. | 15. | Ich sollte mich so verhalten, wie andere es von mir erwarten, um sie glücklich zu machen. |
| 16. | I always want somebody around me. | 16. | Ich möchte immer jemanden um mich haben. |
| 17. | I always want people to show understanding to me. | 17. | Ich möchte, dass die Menschen Verständnis für mich aufbringen. |
| 18. | People should meet each other’s expectations in relationships. | 18. | In Beziehungen sollten Menschen die Erwartungen des jeweils anderen erfüllen. |
| 19. | It is always useful to keep superficial our [*sic*] relationships with others. | 19. | Es ist nützlich, Beziehungen zu anderen oberflächlich zu halten. |
