# Supplementary material for: Measuring dysfunctional interpersonal beliefs: validation of the Interpersonal Cognitive Distortions Scale among a heterogeneous German-speaking sample
Source: BMC Psychiatry. 2023 Sep 27;23:702. doi: 10.1186/s12888-023-05155-3 (PMC10523705; doi:10.1186/s12888-023-05155-3)
Supplement: Supplementary file 2 — Additional file 2: Appendix B. Model fit indices of the EFA that was based on the pooled sample. [file 12888_2023_5155_MOESM2_ESM.docx]

| **Appendix B.** Model fit indices of the EFA that was based on the pooled sample. | | | |
| --- | --- | --- | --- |
| **RMSEA** | **SRMR** | **TLI** | **CFI** |
| .08 | .04 | .80 | .90 |
| *Note.* RMSEA = root mean square error of approximation, SRMR = standardized toot mean residual, TLI = Tucker Lewis index, CFI = comparative fit index. Values < .07 for the RMSEA and < .08 for the SRMR, as well as > .95 for the TLI, and > .95 for the CFI, indicate acceptable threshold levels. | | | |

$\chi^{2}$ (86) = 211.01, *p* < .001
