# Supplementary material for: Measuring dysfunctional interpersonal beliefs: validation of the Interpersonal Cognitive Distortions Scale among a heterogeneous German-speaking sample
Source: BMC Psychiatry. 2023 Sep 27;23:702. doi: 10.1186/s12888-023-05155-3 (PMC10523705; doi:10.1186/s12888-023-05155-3)
Supplement: Supplementary file 3 — Additional file 3: Appendix C. Inter-item correlations of the ICDS. [file 12888_2023_5155_MOESM3_ESM.docx]

| **Appendix C.** Inter-item correlations of the ICDS. | | | | | | | | | | | | | | | | | | | |
| --- | --- | --- | --- | --- | --- | --- | --- | --- | --- | --- | --- | --- | --- | --- | --- | --- | --- | --- | --- |
| Item | 1 | 2 | 3 | 4 | 5 | 6 | 7 | 8 | 9 | 10 | 11 | 12 | 13 | 14 | 15 | 16 | 17 | 18 | 19 |
| 1 | - |  |  |  |  |  |  |  |  |  |  |  |  |  |  |  |  |  |  |
| 2 | **.46** | - |  |  |  |  |  |  |  |  |  |  |  |  |  |  |  |  |  |
| 3 | **.47** | **.56** | - |  |  |  |  |  |  |  |  |  |  |  |  |  |  |  |  |
| 4 | **.49** | **.46** | **.39** | - |  |  |  |  |  |  |  |  |  |  |  |  |  |  |  |
| 5 | **-.16** | -.09 | -.02 | **-.28** | - |  |  |  |  |  |  |  |  |  |  |  |  |  |  |
| 6 | .05 | .01 | -.01 | .12 | .00 | - |  |  |  |  |  |  |  |  |  |  |  |  |  |
| 7 | .10 | .00 | .01 | .12 | .09 | **.56** | - |  |  |  |  |  |  |  |  |  |  |  |  |
| 8 | .10 | .09 | .03 | .12 | **.15** | **.27** | **.41** | - |  |  |  |  |  |  |  |  |  |  |  |
| 9 | -.01 | .08 | .12 | .08 | **.19** | .11 | .13 | **.33** | - |  |  |  |  |  |  |  |  |  |  |
| 10 | **.35** | **.44** | **.41** | **.38** | -.09 | .07 | .03 | -.00 | -.06 | - |  |  |  |  |  |  |  |  |  |
| 11 | -.05 | -.00 | .10 | .03 | .09 | -.09 | .08 | **.15** | **.38** | -.11 | - |  |  |  |  |  |  |  |  |
| 12 | **.35** | **.52** | **.57** | **.30** | -.08 | -.05 | -.07 | .02 | .06 | **.36** | .03 | - |  |  |  |  |  |  |  |
| 13 | **.34** | **.27** | **.37** | **.30** | -.06 | **.24** | **.25** | **.15** | .09 | **.44** | .06 | **.32** | - |  |  |  |  |  |  |
| 14 | **-.19** | -.05 | .01 | -.19 | **.40** | .02 | .09 | .01 | **.23** | -.01 | .13 | .02 | -.09 | - |  |  |  |  |  |
| 15 | .11 | .12 | **.22** | .12 | .03 | -.01 | .04 | **.18** | **.41** | .03 | **.23** | **.24** | .02 | **.28** | - |  |  |  |  |
| 16 | -.02 | .05 | .04 | .08 | **.14** | .07 | .12 | **.19** | **.29** | .02 | **.40** | -.00 | .07 | .09 | **.15** | - |  |  |  |
| 17 | -.11 | .04 | **.16** | -.08 | **.42** | -.01 | .14 | .09 | **.32** | -.01 | **.24** | .09 | .-04 | **.41** | **.24** | **.28** | - |  |  |
| 18 | .03 | -.02 | .02 | .11 | .05 | **.17** | .13 | .11 | **.32** | **.17** | **.17** | -.10 | **.20** | **.19** | **.26** | **.29** | **.21** | - |  |
| 19 | **.33** | **.23.** | **.23** | **.31** | **-.23** | .04 | .00 | -.02 | .10 | **.30** | .11 | .13 | **.38** | **-.21** | .11 | .09 | **-.16** | .12 | - |
| *Note.* Bold numbers indicate significant values (*p* < .05). | | | | | | | | | | | | | | | | | | | |
