# Supplementary figures and images for: Measuring dysfunctional interpersonal beliefs: validation of the Interpersonal Cognitive Distortions Scale among a heterogeneous German-speaking sample
Source: BMC Psychiatry. 2023 Sep 27;23:702. doi: 10.1186/s12888-023-05155-3 (PMC10523705; doi:10.1186/s12888-023-05155-3)

## Slide 1
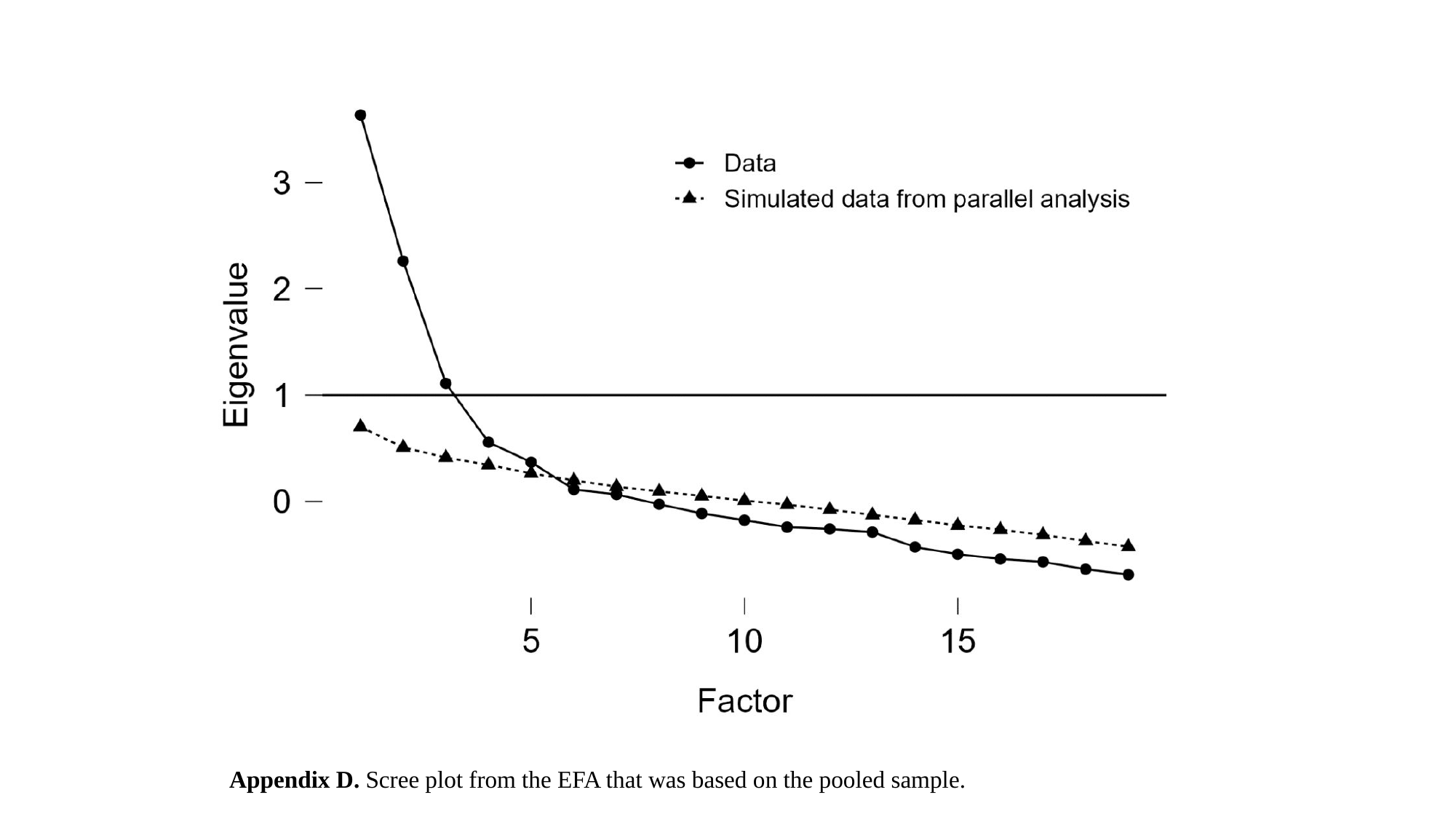

Appendix D. Scree plot from the EFA that was based on the pooled sample.

Supplement: Supplementary file 4 — Additional file 4: Appendix D. Scree plot from the EFA that was based on the pooled sample. [file 12888_2023_5155_MOESM4_ESM.pptx]
